# Genetic Variation at Exon 2 of the MHC Class II *DQB* Locus in Blue Whale (*Balaenoptera musculus*) from the Gulf of California

**DOI:** 10.1371/journal.pone.0141296

**Published:** 2016-01-13

**Authors:** Diana D. Moreno-Santillán, Eileen A. Lacey, Diane Gendron, Jorge Ortega

**Affiliations:** 1 Laboratorio de Bioconservación y Manejo, Escuela Nacional de Ciencias Biológicas, Instituto Politécnico Nacional, Mexico City, Mexico; 2 Museum of Vertebrate Zoology, University of California, Berkeley, California, United States of America; 3 Laboratorio de Ecología de Cetáceos y Quelonios, Centro Interdisciplinario de Ciencias Marinas, Instituto Politécnico Nacional, La Paz, BCS, México; National Cheng-Kung University, TAIWAN

## Abstract

The genes of the Major Histocompatibility Complex (MHC) play an important role in the vertebrate immune response and are among the most polymorphic genes known in vertebrates. In some marine mammals, MHC genes have been shown to be characterized by low levels of polymorphism compared to terrestrial taxa; this reduction in variation is often explained as a result of lower pathogen pressures in marine habitats. To determine if this same reduction in variation applies to the migratory population of blue whales (*Balaenoptera musculus*) that occurs in the Gulf of California, we genotyped a 172 bp fragment of exon 2 of the MHC Class II *DQB* locus for 80 members of this population. Twenty-two putatively functional *DQB* allotypes were identified, all of which were homologous with *DQB* sequences from other cetacean species. Up to 5 putative alleles per individual were identified, suggesting that gene duplication has occurred at this locus. Rates of non-synonymous to synonymous substitutions (ω) and maximum likelihood analyses of models of nucleotide variation provided potential evidence of ongoing positive selection at this exon. Phylogenetic analyses of *DQB* alleles from *B*. *musculus* and 16 other species of cetaceans revealed trans-specific conservation of MHC variants, suggesting that selection has acted on this locus over prolonged periods of time. Collectively our findings reveal that immunogenic variation in blue whales is comparable to that in terrestrial mammals, thereby providing no evidence that marine taxa are subject to reduced pathogen-induced selective pressures.

## Introduction

The major histocompatibility complex (MHC) is a multigene family that plays a fundamental role in the vertebrate immune response [1,2]. Specifically, class II MHC genes code for the cell surface glycoproteins that recognize and bind antigens in order to present them to T-lymphocytes, which initiates the immune response. Class II MHC genes are highly polymorphic in the second exon since this exon codes for the peptide binding region (PBR), which is involved with the recognition and binding to the antigens [1–4]. Therefore, class II MHC genes are typically expected to be subject to strong positive selection, since greater allelic diversity at these loci should be associated with response to a wider range of pathogens [1–3,5]. Diversity of MHC loci is maintained by several mechanisms such as gene conversion, inter-allelic recombination, mating preference, maternal-fetal incompatibility, over-dominant selection and frequency dependent selection [1,2,6]. Such diversity may also be enhanced by gene duplication, which can produce multiple highly similar loci, including functional as well as non-functional (i.e. pseudogenes) MHC sequences. Some of these functional new loci are maintained in the genome for a long period of time as help to detect a wide range of pathogens[6]. As a result, efforts to understand variation at MHC loci must consider the impacts of multiple sources of genotypic diversity.

Presumably as a result of pathogen-mediated balancing selection, MHC genes are typically among the most polymorphic loci in vertebrate genomes [1,2]. Apparent exceptions to this statement occurs in some species of cetaceans (e.g., fin whales, *Balaenoptera physalus*; sei whales, *B*. *borealis*; beluga, *Delphinapterus leucas*), which are characterized by lower levels of MHC variation than most terrestrial mammals surveyed to date [3,7–9]. These data have led to the hypothesis that mammals in marine environments are subject to less intense pathogen pressures than terrestrial taxa [8,9]. Due to the scarcity of MHC studies in marine mammals, support for this hypothesis remains debatable, for example, *Lipotes vexillifer* [10] and *Megaptera novaeangliae* [3] have revealed high levels of variability with 43 and 23 alleles respectively, whilst in *Delphinapterus leucas* eight *DQB* alleles in 233 individuals were reported [9], and in *Balaenoptera physalus* three alleles were found in 36 individuals [7], this being the lowest polymorphism reported in mysticetes. However, patterns of positive selection were comparable to those for terrestrial mammals in all the previous studies in cetaceans[3,7,9–14]; the frequency of non-synonymous substitutions was significantly greater than the frequency of synonymous substitutions in the PBR sites; these results have also been found in MHC class I genes [12]. Thus, the role of the marine environment in shaping selective pressures on MHC genes remains unclear and, as in other species[3,10,11] is likely mediated by a complex variety of factors including current and historical demography as well as behaviorally and ecologically-mediated differences in pathogens exposure. Additionally gene duplication events at the *DQB* locus has been reported in some marine species (*Eschrichtius robustus; Lipotes vexillifer*, *Megaptera novaeangliae; Eubalaena australis*) by finding more than two alleles per individual [3,10,11].

To explore determinants of variation at cetacean MHC loci in greater detail, we characterized patterns of variability and selection at the MHC Class II *DQB* locus in a population of blue whales (*Balaenoptera musculus*) from the Gulf of California, Mexico. This locus was chosen as among mysticetes the *DQB* locus is reportedly more variable than other commonly studied MHC genes such as the *DRB* gene, which seems to be more variable in odontocetes [3,7]). Further, exon 2 of the *DQB* gene encodes the peptide binding region (PBR) of the *DQ* β chain [3,10,11,13].

Although cosmopolitan in distribution, this species is divided into four geographically and genetically distinct subgroups: Northwest Atlantic, Indic Ocean, South Ocean, and East Pacific [15]. After experiencing a severe reduction in size due to commercial whaling, the Pacific subgroup is currently the largest remaining population of this species [16,17]. The Pacific population migrates annually between northern feeding grounds and more southern breeding grounds [17], with a subset of ~ 360 of these animals spending the winter and spring in the Gulf of California, Mexico [18,19]. Given their biology, it seems likely that MHC variation in the Pacific subgroup of blue whales is influenced by a combination of current population structure, demographic history, and exposure to pathogens in a variety of marine habitats. As a first step toward distinguishing between the effects of these factors, we quantified variation at the Class II *DQB* locus for a subset of the individuals overwintering in the Gulf of California. Our analyses generate new insights into patterns of immunogenetic variability in these animals as well as the impacts of selection on cetacean MHC genes over multiple time scales.

## Materials and Methods

### Ethics Statement

Biopsy samples were collected under D. Gendron annual research permits issued by the Secretaría de Medio Ambiente Recursos Naturales y Pesca (DOO 750-00444/99, DOO.0-0095, DOO 02.-8318) from 1997 to 2001, and by the Dirección General de Vida Silvestre, Secretaría de Medio Ambiente y Recursos Naturales (SGPA/DGVS-7000, 00624, 01641, 00560, 12057, 08021, 00506, 09760, 08796, 10646 and 01145) from 2002 to 2012, which now represents the only approved Government institution to issue research permits on endangered species in Mexico. By issuing these annual research permits they approved the methods and the number of blue whale skin-blubber biopsies collection for each year of the permit. A more detailed description of biopsy sampling it could be found in Costa-Urrutia et al. 2013 [18]. All procedures followed guidelines for the use of wild mammals in research established by the American Society of Mammalogists [20].

### Tissue samples

Skin biopsies (35–40 mm) from 80 blue whales (*Balaenoptera musculus*) over-wintering in the Gulf of California, Mexico, were genotyped at the MHC Class II *DQB* locus. Skin samples (5–10 mm) were obtained from Diane Gendron Laniel and colleagues at the Laboratorio de Ecología de Cetáceos y Quelonios, Centro Interdisciplinario de Ciencias Marinas (CICIMAR), Instituto Politécnico Nacional, Mexico. Samples were collected from 1999 to 2012 using a crossbow to shoot a small dart (distance ~ 10 m) at the whale, the tip of dart was designed to secure a skin-blubber biopsy from an individual before falling free so that the dart could be retrieved from the water, this procedure required no direct contact with the study animals [18]. Skin samples were extracted from the dart using sterilized tweezers. An approximately 5 mm^2^ portion of each biopsy was isolated and stored in 70% EtOH at 4°C until sequencing analysis. The remainder of each biopsy was placed in 20% dimethylsulfoxide (DMSO), and then frozen in liquid nitrogen to create a tissue archive for the study population [18]. The identity of each animal biopsied was determined using established photo identification techniques [21,22]; photo identification insured that individual whales were not represented multiple times in our data set.

### DNA extraction and PCR amplification

We examined a 172 bp fragment of exon 2 of the MHC Class II *DQB* locus. Genomic DNA was extracted from tissue samples using the Blood & Tissue DNA Extraction Kit (Qiagen^®^ Inc.) following the protocol of the manufacturer.

Amplification of the *DQB* locus was performed using primers *DQB-F* and *DQB-R* from Murray et al.[9]. Per-sample PCR reaction conditions consisted of 1x PCR Buffer, 0.2mg/ml BSA, 1.5mM MgCl2, 2.4mM dNTPs, 0.5 U of Taq polymerase, 10mM of each primer and 50ng of template DNA in a total reaction volume of 25μl. A touchdown thermo-cycling strategy was used that consisted of initial denaturation at 94°C for 5 min, followed by 30 cycles of denaturing at 94°C for 30 s, initial annealing at 64°C for 30 s (Ta reduced by 2°C after 5, 10, and 15 cycles) and extension at 72°C for 30 s, with a final extension at 72°C for 8 min. PCR products were visualized on 1.5% agarose gels run in 1X TBE buffer and then stained with ethidium bromide (15 μg/ml).

### Cloning and sequencing

To insure that sequencing of the *DQB* exon 2 was limited to a single variant per sequencing reaction, all PCR products of the appropriate size (225 bp) were cloned using the TOPO^®^ TA Cloning^®^ kit (Invitrogen Inc.) following the instructions of the manufacturer. Sequencing of eight clones per PCR reaction is expected to have a high probability (*p* = 0.992) of detecting both alleles in heterozygous individuals [23] and thus we amplified a minimum of eight positive clones per animal using the vector specific primers M13R and M13F and following the protocol of the manufacturer. Amplified products were visualized on agarose gels as described above; products with the correct insert size were cleaned with ExoSap and then sequenced using the ABI BigDye terminator Cycle Sequencing Kit (ABI, Inc.). All cloning products were sequenced in the forward and reverse directions on an ABI3130 automated sequencer.

### Identification of MHC alleles

All sequences were aligned and edited using the program Geneious .5.6.6 [24]. To determine if stop codons or other evidence of possible pseudogenes were present, sequences were translated into the corresponding amino acid sequences using Geneious [24]. Variability and polymorphism in amino acid sequences were calculated using the Wu-Kabat (W-K) statistic measure [25], with highly polymorphic sites defined as those with greater than twice the mean W-K score.

At the nucleotide level, we defined sequence variants as distinct alleles following the criteria of Kennedy et al. [26]: variants were considered to be distinct alleles only if they were present in three clones from the same individual or in at least one clone from two different individuals. To confirm that identified alleles were from the *DQB* locus, we performed BLAST searches [27] using the National Center for Biotechnology (NCBI) database.

Nomenclature for the *DQB* alleles identified for *B*. *musculus* followed Kennedy et al.[26]; each allotype was given the prefix *Bamu* (corresponding to the two first letters of the genus and the species names) and the suffix GC, which represents the Gulf of California locality where the tissues samples were collected [3]. *DQB* sequences generated during this study were accessioned into GenBank as numbers: KJ179618 to KJ179639.

### Detecting selection

Evidence of selection at the molecular level was assessed using three distinct procedures. First, we calculated rates of synonymous (d_S_) and non-synonymous (d_N_) base pair substitutions (d_N_/ d_S_, denoted here as ω) for all codons as well as for the subset of codons corresponding to the PBR of this exon. Ratios greater than one (ω > 1) indicate an excess of non-synonymous substitutions relative to neutral expectations and such values are typically interpreted as evidence of positive selection [28,29]. Ratios were calculated following the method of Nei-Gojobori and using the Jukes-Cantor correction [28], as implemented in DNAsp 5.1 [30]. Within exon 2, we used comparisons with sequences from the human homolog of the *DQB* locus [31] to identify the subset of codons corresponding to the putative PBR in sequences from *B*. *musculus*. We calculated values of ω for the putative PBR following the above procedure. Significance tests of differences between ω values were conducted using the Z-test contained in MEGA 5.2 [32].

As a second test of neutrality, we calculated Tajima’s D for the study population using DNAsp5.11 [30]. This test is based on differences between estimates of nucleotide diversity (π) and the number of segregating sites (Θ). For neutral loci *D* = 0; a negative value for *D* can be interpreted as evidence of purifying selection, while a positive value for *D* can be interpreted as evidence of positive selection [33].

Finally, as a third test of neutrality, we examined several models of molecular evolution using the CODEML subroutine contained in PAML XI [34]. This procedure is more sensitive in detecting selection at the molecular level than previous analyses because it to uses a maximum likelihood ratio test (LRT), to compare a null model (no selection) against multiple alternative models that incorporate selection [29]. We tested six distributional models which allow different patterns of selection: M0 (one ratio), M1 (neutral), M2 (positive selection), M3 (discrete), M7 (β model) and M8 (β and ω) [29,35]. Three likelihood comparisons were performed between null and alternative models that allow selection: M0 vs M3, M1 vs M2 and M7 vs M8 [29,35]. The significance of each comparisons was assessed using a Chi-square test [35]. Models M2 and M8 allow identification of individual sites that are subject to selection, we used these models to calculate posterior probabilities for each site using Bayes empirical Bayes (BEB) [29].

### Phylogenetic analyses

To assess evidence for trans-species conservation of *DQB* alleles, we constructed a phylogenetic tree that included the *DQB* sequences generated in this study plus those from 16 other species of cetaceans obtained from GenBank (Table 1), *DQB* alleles from *Bos taurus* were used as an out-group. A neighbor joining (NJ) tree reconstruction was conducted by using the matrix of distances compound as implemented in PAUP* 4.0b10 [36], genetic distances were adjusted using the best-fit model of sequence evolution determined by using hierarchical likelihood ratio tests based on the Akaike Information Criterion (AIC) as executed in JModeltest 3.7 [37]. These analyses revealed that the nucleotide substitution model F8+I+G (gamma distribution: 0.3200; invariable sites: 0.3040; base composition: A = 0.2263, C = 0.2793, G = 0.3730 and T = 0.1215) best described our data set. The branch support was assessed with 1,000 bootstrap replicates. Likewise phylogenetic reconstruction was performed using Bayesian analyses with Mr. Bayes 3.2.5 [38], the runs consisted of four heated and one cold Markov chains (heating = 0.20), we sampled every 20,000 generations, until the stationary distribution indicated by an average of the standard deviation was <0.01. Posterior probability was calculated by computing the majority rule consensus tree from the remaining trees. An additional measure of relationships between *DQB* alleles from *B*. *musculus* and other cetaceans, we constructed a median-joining haplotype network using Network 4.6 [39], which is based on the minimum number of mutations between alleles [39].

**Table 1:** Species and accession numbers for *DQB* loci sequences downloaded from GeneBank, included in the phylogenetic analysis.

Order	Family	Species	Gene Bank Accesion numbers
**Cetacea**			
	Balaenidae	*Balaena mysticetus*	Bamy92004-DQB*2 (DQ354624)
		*Eubalaena australis*	Euau-DQB*4C (DQ354633)
	Balaenopteridae	*Balaenoptera acutorostrata*	Baac-a (AB164201)
		*Balaenoptera bonaerensis*	Babo-c (AB164204)
		*Balaenoptera musculus*	Bamu-a (AB16420)
		*Balaenoptera physalus*	BaphGC-DQB (DQ300261)
		*Megaptera novaeangliae*	MenoSEA-DQB*20c (DQ354661)
	Delphinidae	*Cephalorhynchus hectori*	CeheNI01-DQB*2 (DQ354629)
		*Delphinus delphis*	Dede-a (AB164220)
		*Globicephala macrorhynchus*	Glma-b (AB164227)
		*Orcaella brevirostris*	Orbr-a (AB164223)
		*Tursiops truncatus*	Tutr-a (AB164221)
	Eschrichtiidae	*Eschrichtius robustus*	EsroWa002-DQB*2 (DQ354636)
	Iniidae	*Lipotes vexillifer*	Live-DQB*4 (AY177153)
	Monodontidae	*Delphinapterus leucas*	Dele-DQ beta-0201 (U16989)
		*Monodon monoceros*	Momo-DQB-0201 (U16991)
**Artiodactyla**			
	Bovidae	*Bos taurus*	DQB-HH13A (AF078159)
		*Bos taurus*	BoLA-DQB*Q-A06 (AY444365)

## Results

### *DQB* variation

**Fig 1:**
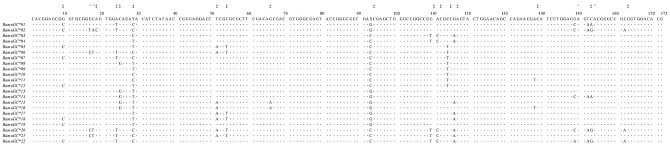
Nucleotide alignment of *DQB* alleles from *B*. *musculus*. Parsimony sites of two variants are represented with ǂ and three variants are represented with ^. The identity with the consensus sequence is indicated by a dot (·).

A total of 22 distinct alleles of exon 2 of the *DQB* locus was identified from the 80 blue whales examined (Fig 1); these sequences had assigned GenBank accession numbers KJ179618-KJ179639‏. No insertions, deletions or stop codons were detected in our study, suggesting that they may represent expressed copies of the *DQB* locus in our study population.

A total of 757 cloned PCR products (mean = 9.8 **±** 2.9 products per individual), were sequenced. More than two *DQB* alleles were found in 36 (45%) of the surveyed animals (mean 4.83±1.11 alleles per individual, range = 1–5), suggesting that members of the population possessed multiple copies of this *DQB* locus.

**Fig 2:**
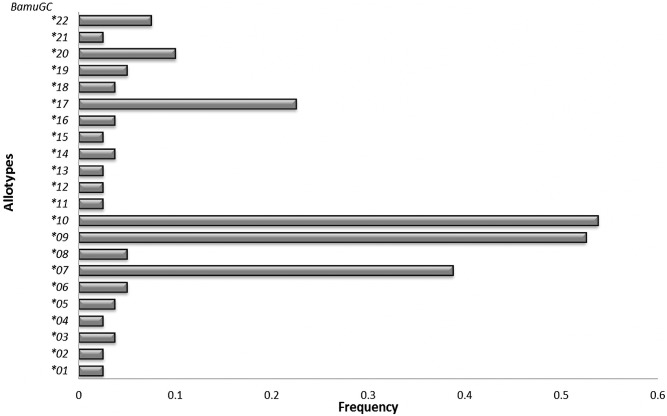
Variation in allotype frequencies in *B*. *musculus* from the Gulf of California.

A BLAST search indicated that the allotypes detected in our population displayed 90% to 99% concordance with exon 2 of the *DQB* locus from other cetacean, with sequences from humpback whales (*Megaptera novaeangliae*) being the most similar[13]. Variation in allotypes frequencies for the study population is presented in Fig 2.

**Fig 3:**
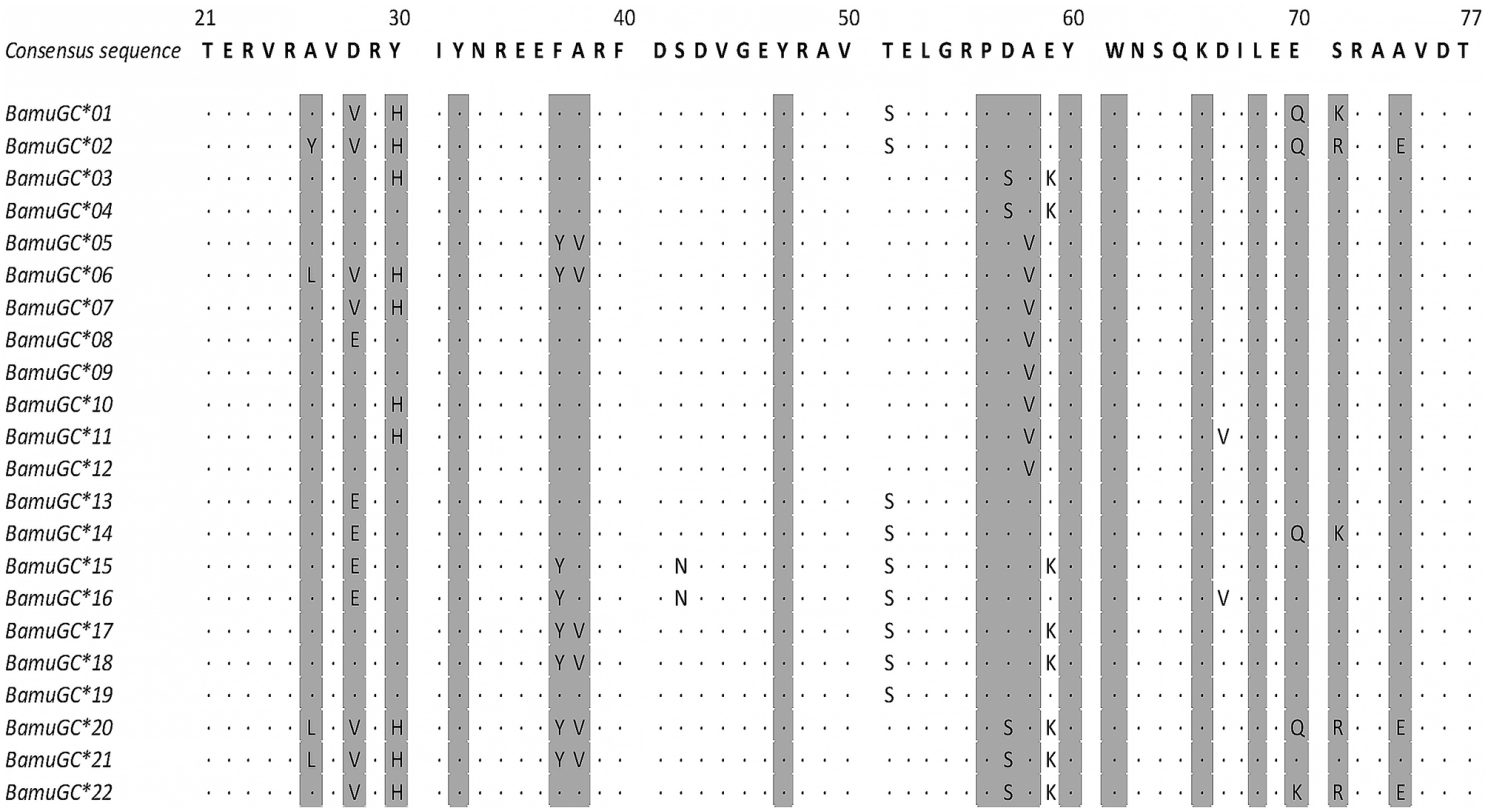
Amino acid alignment of *DQB* allotypes from *B*. *musculus*. The alignment was based on the nucleotide sequences (Fig 1). The identity with the consensus sequence is indicated by a dot (·). Putative PBR sites are shadowed and were assigned according to the three dimensional structure of human and beluga MHC-II molecule structure [9, 31]. The numbers above the consensus sequence correspond to amino acid positions of the human class II beta chain structure [31].

**Fig 4:**
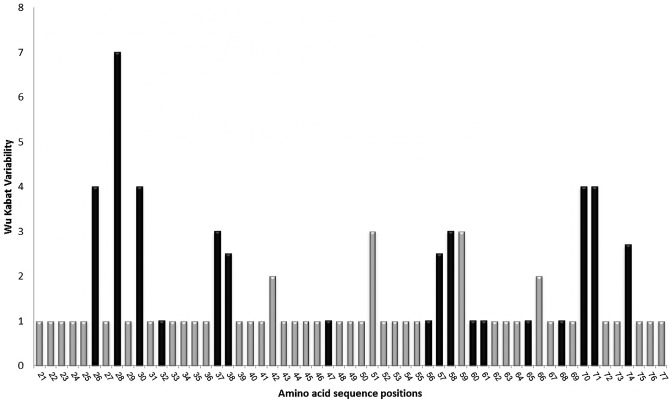
Amino acid variability plot Wu and Kabat. Amino acid variability plot Wu and Kabat for the amino acid residues of the Blue whale *DQB* allotypes. Darker bars indicate the putative PBR.

At the amino acid level, our sequenced fragment contained 57 amino acids of *DQB*. Fig 3 shows the predicted sequence of each of our allotypes along with the putative sites forming the PBR of exon 2, which is comprised within the amino acids 6 to 54. Comparisons of all allotypes revealed 21 variable sites, 17 (71.4%) of which occurred in the PBR region; these variable sites coded for 22 different amino acid sequences. A plot of amino acid variability revealed that despite the large number of variable sites, the variation of each residue is relatively low among allotypes (Fig 4). The Wu-Kabat score for our data was 2.0 consequently highly polymorphic sites are defined as those with Wu-Kabat scores greater than 4. Using this criterion, 5 sites (7.01%) were identified as highly polymorphic.

### Evidence of selection

We obtained a positive value of the Tajima’s D (D = 1.078), however statistics calculations did not show a significant departure from neutrality at *DQB* exon 2 as indicated by the *P* value (*P*> 3.05).

The number of non-synonymous substitutions was significantly greater than the number of synonymous substitutions (ω > 0; *p* < 0.01) in the entire 172 bp *DQB* sequence. Otherwise comparisons of ω for the PBR and non-PBR portions of exon 2 revealed that 10 (71.4%) of the 14 amino acid changes detected occurred in the PBR. Within the PBR, ω was significantly greater than one (ω = 93; *p >* 0.01), in contrast, ω did not differ significantly from neutral expectations (ω = 0.04; *p* > 0.05) for the non-PBR portion of the exon (Table 2). PAML analyses revealed that models of nucleotide evolution that incorporate selection performed significantly better than models that do not allow selection (Table 3).

**Table 2:** Rate of synonymous and non-synonymous substitutions in blue whale *DQB* sequences, at the PBR, non-PBR sites and all the sequences.

Positions	Codons	d_N_	d_S_	ω	*P*
PBR	17	0.093 (0.027)	0.001 (0.001)	93	<0.01*
Non-PBR	40	0.009 (0.006)	0.014 (0.014)	0.64	> 0.05
All	57	0.056 (0.015)	0.015 (0.012)	3.73	< 0.01*

ω = (d_N_/ d_S_); Significant *P* values obtained by Z-test are mark with a *. The numbers in parenthesis correspond to the standard errors, obtained through 1,000 bootstrap replicates.

**Table 3:** Summary of the likelihood-ratio test of exon 2 of the *DQB* locus in *B*. *musculus*.

Models compared	*d*.*f*	Test statistics	*P* value
M2a vs M1a	2	59.04	*<0*.*001*
M3 vs M0	4	92.05	*<0*.*001*
M8 vs M7	2	59.05	*<0*.*001*

*df* degrees of freedom; Test statistics was computed as 2(*L*_*b*_*- L*_*a*_*)*. *L*_*a*_ and *L*_*b*_ are log-likelihood values for each of the nested models compared.

Specifically, model M2a identified 13 codons that appear to have experienced significant selection; model M8 identified the same 13 codons plus one additional codon as subject to selection. Eleven of the sites identified by both models occurred in the putative PBR region (Table 4). These findings suggest that within our study population, exon 2 of the *DQB* locus shows significant departures from neutrality with evidence of selection at multiple sites occurring within the functionally important PBR.

**Table 4:** Results of maximum likelihood models of exon 2 of the *DQB* locus in *B*. *musculus*.

Model code	*P*	Log-likelihood	Parameter estimates	Positively selected sites
M0(one radio)	1	-582.380683	ω = 3.90940k = 1.27787	None
M1a(Nearly neutral)	1	-567.590140	p_0_ = 0.69220, p_1_ = 0.30780, K = 0.86424 ω_0_ = 0, ω_1_ = 1	Not allowed
M2a(Positive selection)	3	-538.070207	p_0_ = 0.68594, p_1_ = 0.07889, p_2_ = 0.23517, K = 1.29556ω_0_ = 0, ω_1_ = 1, ω_2_ = 18.57525	**6A**, **8V**, **10H**, **17F**, **18A**, 31S, *37D*, **38A**, **39E**, 46D, **50Q**, **51K**, *54A*
M3 (discrete)	5	-536.350896	p_0_ = 0.73468, p_1_ = 0.17464, p_2_ = 0.09068, K = 1.28190ω_0_ = 0, ω_1_ = 10.74687, ω_2_ = 32.3886	Not analyzed
M7 (β)	2	-567.595779	p = 0.00500, q = 0.01170 K = 0.86299	Not allowed
M8 (β and ω)	4	-538.070384	p_0_ = 0.76454, p_1_ = 0.23546, p_2_ = 0.005, q = 0.048, ω = 18.53364 K = 1.29571,	**6A**, **8V**, **10H**, **17F**, **18A**, 22S, **31S**, **37D**, **38A**, **39E**, 46D, **50Q**, **51K**, **54A**

Positive selected sites were identified using empirical Bayes procedure. Sites inferred under selection at the 99% level are listed in bold, and those at the 95% level are in italics, the putative PBR sites are underlined. For models 7 and 8, *p* and q are the shape parameters of β function. (*P*) is the number of parameters in the ω distribution, (*K*) the estimated transition/transversion rate ratio, (ω) the selection parameter and (p_n_) the proportions of sites that fall into the ωn site class.

### Phylogenetic relationships among *DQB* alleles

**Fig 5:**
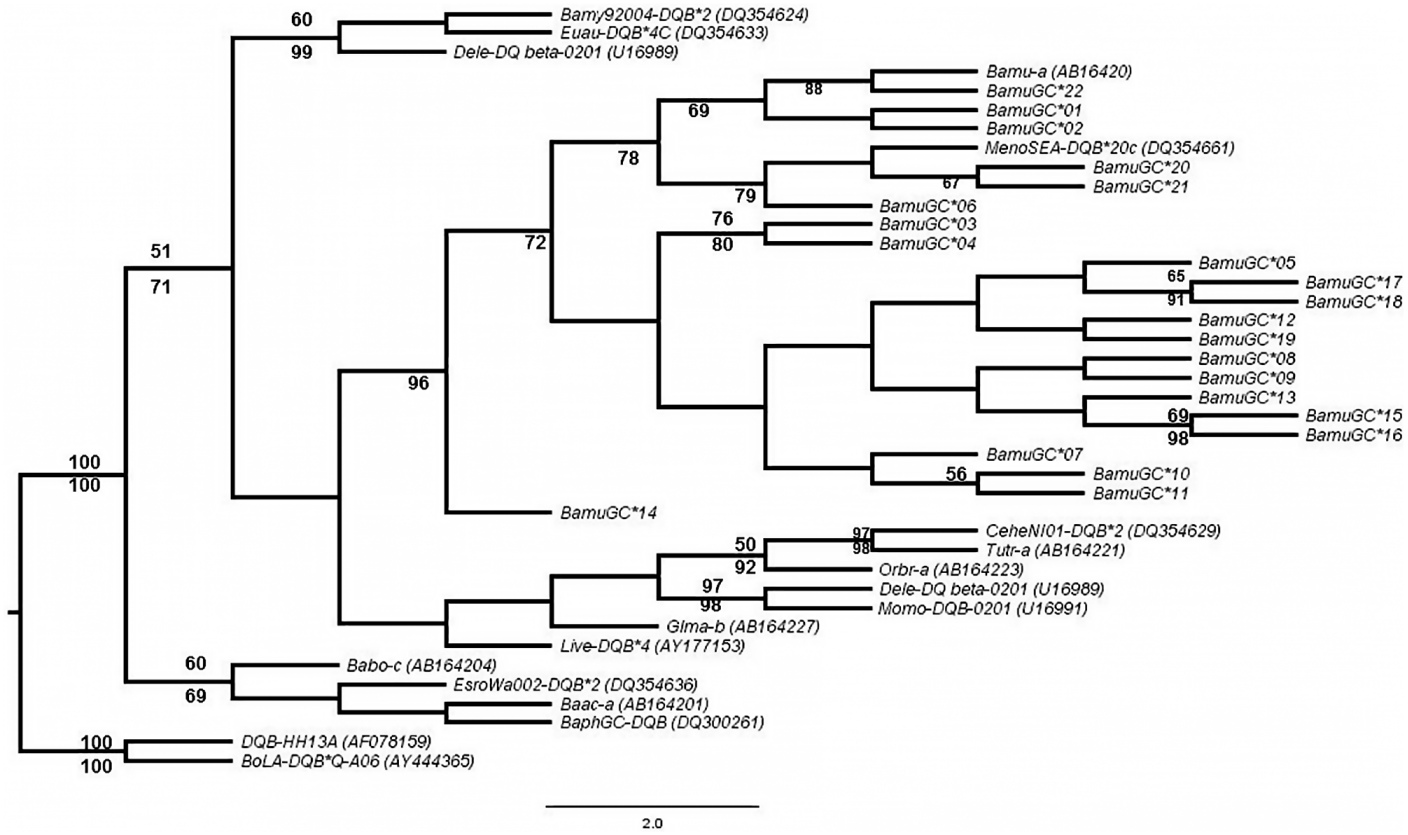
Phylogenetic likelihood tree reconstruction of the exon 2*DQB* of *B*. *musculus* and other cetacean, with *Bos taurus* as an out-group. Sequences of other cetaceans were downloaded from GeneBank database (nomenclature and accession numbers are presented in Table 1). Bootstrap values above 50% from the Neighbor-joining analysis are presented above respective branches whilst Bayesian posterior probabilities above 60% are shown below its respective branches.

**Fig 6:**
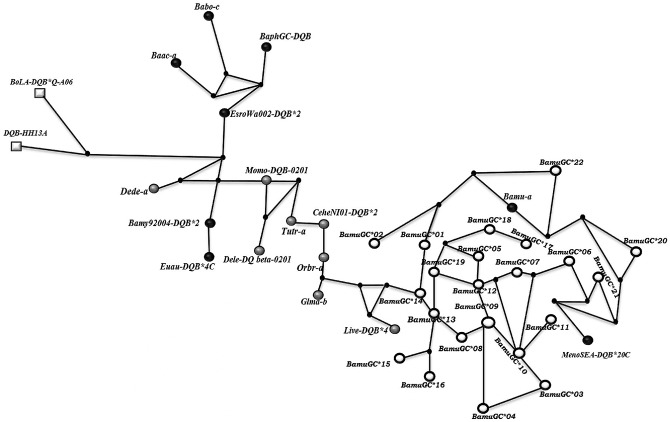
Median-Joining haplotype network of the exon 2 *DQB* locus of *B*. *musculus* and other cetaceans. Median-Joining haplotype network of the exon 2 *DQB* locus of *B*. *musculus* and other cetaceans with *Bos taurus* as the outgroup (white squares). The homology sequences of Mysticetes and Odontocetes were downloaded from GeneBank database (Nomenclature and accession numbers are presented in Table 1). Gray circles represent odontocete’s alleles, black circles correspond to mysticete alleles and white circles correspond to Blue whale alleles obtained in this study.

Phylogenetic analyses of *DQB* alleles revealed that all cetacean sequences included in our analyses formed a strong well supported monophyletic group distinct from *Bos taurus* (Fig 5), within the examined cetaceans, suborders Odontocete and Mysticete were not reciprocally monophyletic with respect to the *DQB* alleles, nor did *DQB* alleles of *B*. *musculus* form a monophyletic clade with respect to other cetacean species of *Balaenopteridae* family (Fig 5). These analyses revealed evidence of trans-specific maintenance of *DQB* alleles between *B*. *musculus* and *M*. *novaeangliae* (Fig 5). Haplotype networks constructed from these data, revealed that some sequences for *B musculus* were more similar to those found in *M*. *novaeangliae* than to other alleles detected for *B*. *musculus* (Fig 6). Thus, within cetaceans, exon 2 of the *DQB* locus appears to be characterized by trans-specific maintenance of alleles.

## Discussion

Our analyses revealed substantial variation at exon 2 of the *DQB* locus within the population of *B*. *musculus* in the Gulf of California, although, the number of variants per individual detected revealed the presence of multiple copies of this locus. We followed the criteria of MacManes et al. [23], wherein it is suggested that with 8 clones per individual both alleles of an heterozygote individual might be detected with a probability of 0.992. However this assumption was made under a probabilistic model with binomial distribution, hence, as *DQB* locus presents multiple copies it is important to consider that the number of variants might be underestimated as a result of a small number of clones analyzed per individual. Analyses of all detected alleles revealed significant departures from neutrality. Likelihood ratio tests indicated that multiple sites within the functionally important peptide binding region of exon 2 appear to have been subject to selection. Our data are consistent with previous studies in revealing evidence of both gene duplication and positive selection on the *DQB* gene.

### Evidence of selection

Class II MHC loci are typically thought to be subject to strong positive selection due to their role in detecting and responding to pathogens[1–4]. Our findings are consistent with expectation in that we found significant nucleotide-level evidence of selection on exon 2 of the *DQB* locus in the population of blue whales in the Gulf of California. Both rates of non-synonymous to synonymous substitutions and analyses of different models of molecular evolution indicated significant departures from neutrality, particularly within the functionally important protein binding region (PBR) of this exon. The *DQB* locus in our study population appears to be subject to selection at the nucleotide level.

Comparisons of *DQB* alleles from our study animals and other cetaceans revealed evidence of trans-specific preservation of alleles, particularly between blue and humpback whales. Similar trans-specific conservation of MHC alleles has been reported for a variety of mammalian species including non-human primates [40], bank voles [41], and tuco-tucos [5] as well as multiple species of marine mammals [7,10,13,14,42]. Trans-specific conservation of MHC alleles is typically thought to reflect long-term selection for MHC variants that are beneficial in responding to pathogens. Collectively, these data and our finding regarding nucleotide level variation suggest that the *DQB* locus in blue whales from the Gulf of California have been subject to selection over multiple scales.

### MHC variation in marine mammals

In contrast to our findings, studies of a number of species of cetaceans have reported limited variability at MHC loci [3,7–9,11], leading to the hypothesis that individuals in marine environments experience reduced pathogen pressure compared to those in terrestrial habitats. Support for this idea, however, is inconsistent. For example, in addition to our data for blue whales, marked MHC variability has been reported for Chinese river dolphins (*L*. *vexillifer*: 33 functional alleles in 18 individuals [10]), and Atlantic bottlenose dolphins (*T*. *truncates*: 27 functional alleles in 48 individuals [43]. Further, while our data indicate that *B*. *musculus* from the Gulf of California are characterized by considerable MHC variation, the population of fin whales (*B*. *physalus*) from this locality displays low levels of MHC variation [7], suggesting that environmental conditions alone do not explain differences in immunogenetic variation among marine mammals. Additional studies—including both genetic analyses of additional cetacean species and quantitative assays of pathogen exposure across multiple environments—are needed to untangle relationships between marine habitats, pathogen pressures, and interspecific differences in variability at MHC loci.

Our analyses also revealed evidence of multiple copies of the *DQB* locus per individual in our study population. None of the alleles detected contained insertions, deletions, or stop codons, suggesting that some of these copies might represent functional sequences. We consider that our findings suggest that one or more gene duplication events have resulted in multiple functional copies of *DQB* locus, this assumption supports the hypothesis that cetacean *DQB* sequences are paralogous genes separated by gene duplication events, which is thought to be one of the major forces for MHC evolution [44]. Gene duplication at the *DQB* locus has been reported for several other cetacean species (e. g., *Lipotes vexillifer* [10], *Megaptera novaeangliae*, *Eubalaena australis* [3] and *Neophocaena phocaenoides* [11]) as well as multiple species of terrestrial mammals (e.g., *Bos indicus* [45], *Cervus elaphus* [46], *Bubalus bubalis* [47], *Mastomys natalensis* [48]), therefore this might be a relatively common occurrence at this locus. Gene duplication is a continual process in evolution that produces new genes and functions according to the birth-death-model, therefore this is one of the evolutionary mechanisms that maintains the high polymorphism in the MHC genes [6,41,49,50] In this regard, we assume that natural selection has been maintaining functional duplicated loci in MHC genes over time, as these new variants might be enabling an increment in the recognition and union of a wider range of pathogens, giving a better capacity of immune response. It must be of consideration that duplication events affect the estimates of selection, i.e. having multiple functional genes would tend to increase variability and divergence among alleles, and therefore we could be potentially overestimating evidence of selection.

#### Influence of demography

One factor that may contribute to interspecific differences in immunogenetic variability among marine mammals is demography. For example, the population of fin whales in the Gulf of California is resident in that area and is isolated from a population of conspecifics [7]. The Gulf of California population experienced a significant bottleneck during the past century and was reduced to an estimated 400–800 individuals [7]. This combination of historical reduction in population size and limited current gene flow is thought to have contributed to limited genetic variability, including variability at MHC genes, in this population. In contrast, the study population of blue whales is migratory, traveling annually between high latitude feeding grounds and low latitude breeding sites [17]. Although this population experienced a historical reduction in size due to commercial whaling—reaching a low of 1400 individuals in 1976 –it is thought that this population has recovered between 75–100% of the initial estimated abundance [51]. While the relative genetic impacts of the historical bottlenecks experienced by these populations have not been assessed, the differences in current demography between fin and blue whales in the Gulf of California are consistent with the apparently greater genetic variability in the latter species. More generally, comparisons of these populations suggest that current and historical demography may play important roles in shaping immunogenetic diversity in marine mammals.

## Conclusions

Our analyses of variation at exon 2 of the *DQB* locus in blue whales from the Gulf of California revealed levels of immunogenetic variability comparable to those of many terrestrial mammals. Further, our data revealed evidence of selection on this exon at both the nucleotide and trans-specific levels, thereby contradicting the hypothesis that marine taxa are subject to less intense selective pressures than terrestrial mammals. Instead, we suggest that differences in current and historical demography may better explain the differences in immunogenetic variability evident among marine mammals. Future studies of MHC variation in blue whales and other marine taxa will benefit from exploring differences in pathogen-induced selective pressures as a function of both habitat and variation in current and historical demography.
